# Colonic phytobezoar in a child

**DOI:** 10.1002/jpr3.12146

**Published:** 2024-11-11

**Authors:** Alexandra Oxford, Carine Stearman, John F. Pohl

**Affiliations:** ^1^ Division of Pediatric Gastroenterology University of Utah Salt Lake City Utah USA

**Keywords:** bezoar, colon, gastroenterology, obstruction, pediatric

## Abstract

Gastric bezoars and small bowel bezoars are uncommon in the pediatric population, and colonic bezoars are even rarer. We present the case of a 9‐year‐old female who presented with acute abdominal obstruction symptoms and a diagnosis of colonic phytobezoar. The phytobezoar was removed via endoscopic intervention. This case is important as it demonstrates that colonic bezoars can occur in children, may not be amenable to oral laxative or enema therapy, and may require endoscopic removal.

## 1 INTRODUCTION

A bezoar is an undigested mass of material that accumulates in the gastrointestinal tract and leads to obstruction. Such bezoars can consist of hair (trichobezoar), medication (pharmacobezoar), milk (lactobezoar), and vegetable matter (phytobezoar).^1^

Although bezoars are relatively rare clinical presentations, bezoars of the small intestine and stomach are more common than colonic bezoars.^2^ We present the case of a child who presented with an acute abdominal obstruction due to a colonic phytobezoar.

## 2 PRESENTATION OF CASE

**Figure 1:**
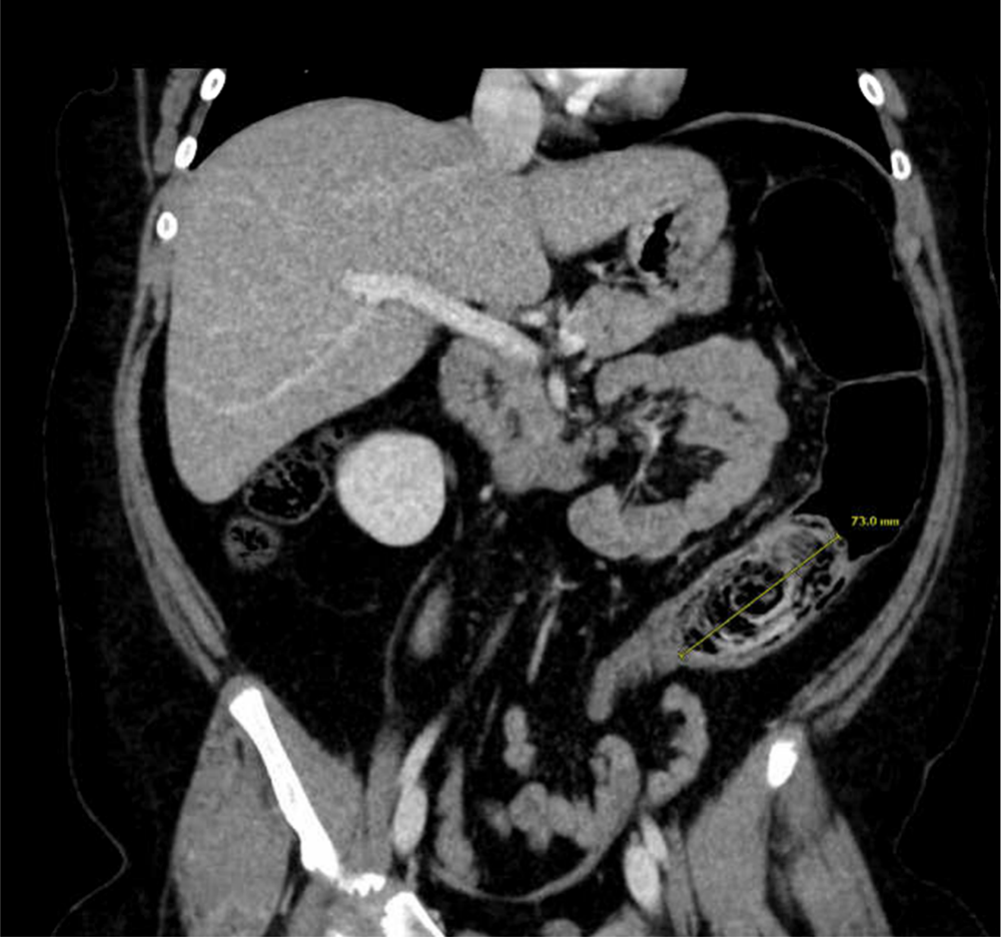
Coronal view of patient's abdomen demonstrating an obstructive mass consistent with a colonic bezoar in the mid to distal descending colon.

**Figure 2:**
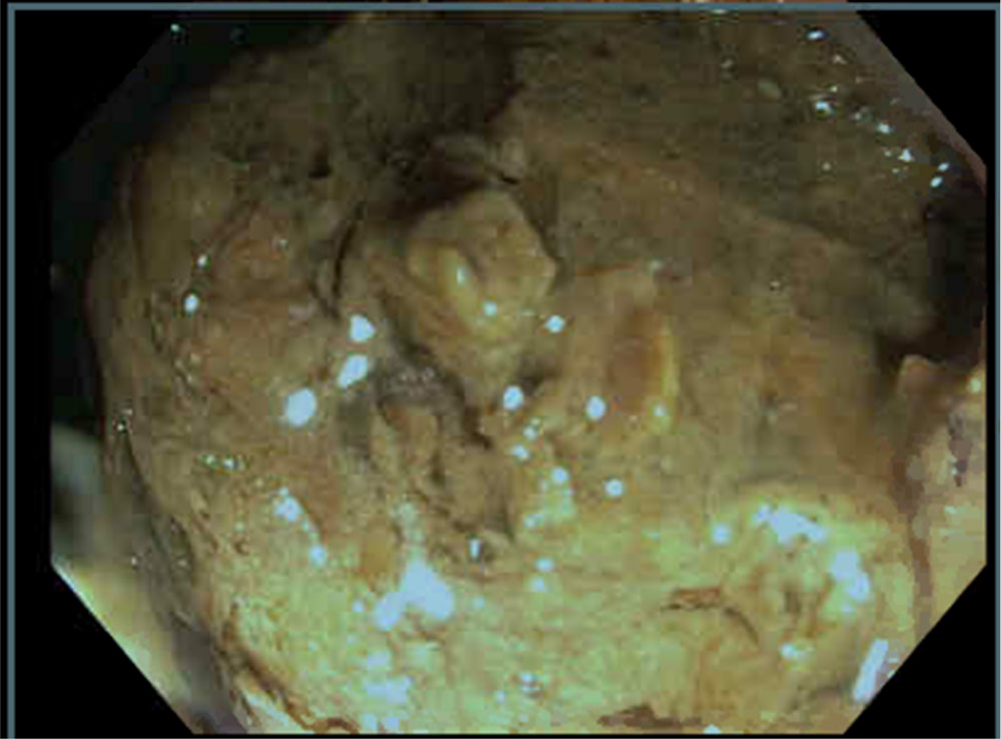
Colonoscopy view of phytobezoar obstructing the colon.

A previously healthy 9‐year‐old female with no known medical history was transferred to the emergency department from an outside hospital with acute onset, severe abdominal pain and nonbloody, nonbilious vomiting after eating at a restaurant. The abdominal pain was located in the lower left quadrant. An abdominal computed tomography scan demonstrated colonic dilation with a lamellated fecal‐containing structure believed to be a bezoar measuring approximately 7 cm in length located in the mid to distal descending colon causing a colonic obstruction with colonic dilation present proximal to the obstruction (Figure 1). The patient had no history of constipation, but she had a dietary history of consuming raw vegetables, particularly broccoli. The obstruction could not be relieved with serial enemas or large‐volume nasogastric polyethylene glycol 3350 therapy over a 24‐h period. Thus, the patient underwent therapeutic colonoscopy that demonstrated a phytobezoar approximately 40–45 cm from the anus (Figure 2) that was completely obstructing the colon. It was removed using a combination of rat‐tooth forceps, polypectomy basket, and transparent endoscopic cap to increase the suction pressure. After the phytobezoar was removed, there was an immediate release of fecal material from behind the obstructed area. The patient tolerated the procedure well and was able to eat afterward with no emesis. She was discharged home without abdominal pain and vomiting.

## 3 DISCUSSION

Phytobezoars are composed of plant matter and are further classified as either fiber or seed‐based concretions. Specific foods commonly implicated in bezoar formation are those high in lignin and cellulose (oranges, persimmons, and potato skins) and the stems of cruciferous vegetables (broccoli, cabbage, and Brussels sprouts). Phytobezoars are typically found proximal to the ileocecal valve.^3^, ^4^ However, patients with predisposing conditions such as prior abdominal surgery, muscle atrophy, cerebrovascular disease, or delayed colonic motility have an increased risk of colonic phytobezoar involvement owing to the risk of pre‐existing colonic stricturing or impaired eating ability. Theoretically, such clinical scenarios are more common in adults than in the pediatric population.^4^, ^5^

To our knowledge, this is the first case report describing a colonic phytobezoar attributable to *cruciferous* vegetable fibers in a pediatric patient. A literature search of PubMed revealed only two relevant pediatric case reports describing colonic bezoar formation attributed to tomato ingestion in a 16‐month‐old patient and a rectal phytobezoar composed of sunflower seeds at 6 years old.^6^, ^7^ The latter of these publications is most similar to the case reported here; however, as proposed by Manatakis et al., seed bezoars may be better classified as a disparate diagnosis to fiber‐based phytobezoars, as the pathophysiology leading to formation is distinct.^4^

## 4 CONCLUSION

Our case of a colonic cruciferous phytobezoar in a pediatric patient with acute abdominal obstruction represents a unique contribution of an underreported condition to the pediatric gastroenterology literature, with the most similar case studies published over 20 years ago. It should be remembered that colonic phytobezoars are rare but may present as an acute abdominal obstruction in children, and endoscopic therapeutic removal of the obstructing colonic phytobezoar is imperative. Surgical removal of colonic phytobezoars should be reserved only for colonic bezoars that cannot be removed endoscopically or for bezoars associated with colonic ischemia.^8^

## CONFLICT OF INTEREST STATEMENT

The authors declare no conflicts of interest.

## References

[1] Iwamuro M , Okada H , Matsueda K , et al. Review of the diagnosis and management of gastrointestinal bezoars. World J Gastrointest Endosc. 2015;7:336‐345. 25901212 10.4253/wjge.v7.i4.336 PMC4400622

[2] Law GW , Lin D , Thomas R . Colonic phytobezoar as a rare cause of large bowel obstruction. BMJ Case Rep. 2015;9:bcr2014208493. 10.1136/bcr-2014-208493 PMC4401910 25858930

[3] Kramer SJ , Pochapin MB . Gastric phytobezoar dissolution with ingestion of Diet Coke and cellulase. Gastroenterol Hepatol (NY). 2012;8:770‐772. PMC3966177 24672417

[4] Manatakis DK , Acheimastos V , Antonopoulou MI , Balalis D , Korkolis DP . Gastrointestinal seed bezoars: a systematic review of case reports and case series. Cureus. 2019;11:e4686. 31333915 10.7759/cureus.4686 PMC6636697

[5] Eng K , Kay M . Gastrointestinal bezoars: history and current treatment paradigms. Gastroenterol Hepatol (NY). 2012;8:776‐778. PMC3966178 24672418

[6] Steinberg R , Schwarz M , Gelber E , Lerner A , Zer M . A rare case of colonic obstruction by “cherry tomato” phytobezoar: a simple technique to avoid enterotomy. J Pediatr Surg. 2002;37:794‐795. 11987104 10.1053/jpsu.2002.32290

[7] Sawnani H , McFarlane‐Ferreira Y . Proctological crunch: sunflower‐seed bezoar. J La State Med Soc. 2003;155:163‐164. 12873104

[8] Lau C , Myers J , Brown M . Large bowel obstruction secondary to a colonic bezoar: a case report. Am J Gastroenterol. 2010;105:S322.

